# The Evolutionary History of the Rediscovered Austrian Population of the Giant Centipede *Scolopendra cingulata* Latreille 1829 (Chilopoda, Scolopendromorpha)

**DOI:** 10.1371/journal.pone.0108650

**Published:** 2014-09-24

**Authors:** Jan Philip Oeyen, Sebastian Funke, Wolfgang Böhme, Thomas Wesener

**Affiliations:** 1 Department Arthropoda, Zoological Research Museum Alexander Koenig, Bonn, Germany; 2 Department of Ophthalmology, University Medical Center of the Johannes Gutenberg-University, Mainz, Germany; Institute of Biochemistry and Biology, Germany

## Abstract

The thermophilous giant centipede *Scolopendra cingulata* is a voracious terrestrial predator, which uses its modified first leg pair and potent venom to capture prey. The highly variable species is the most common of the genus in Europe, occurring from Portugal in the west to Iran in the east. The northernmost occurrences are in Hungary and Romania, where it abides in small isolated fringe populations. We report the rediscovery of an isolated Austrian population of *Scolopendra cingulata* with the first explicit specimen records for more than 80 years and provide insights into the evolutionary history of the northernmost populations utilizing fragments of two mitochondrial genes, COI and 16S, comprising 1,155 base pairs. We test the previously proposed hypothesis of a speciation by distance scenario, which argued for a simple range expansion of the species from the southeast, via Romania, Hungary and finally to Austria, based on a comprehensive taxon sampling from seven countries, including the first European mainland samples. We argue that more complex patterns must have shaped the current distribution of *S. cingulata* and that the Austrian population should be viewed as an important biogeographical relict in a possible microrefugium. The unique haplotype of the Austrian population could constitute an important part of the species genetic diversity and we hope that this discovery will initiate protective measures not only for *S. cingulata*, but also for its habitat, since microrefugia are likely to host further rare thermophilous species. Furthermore, we take advantage of the unprecedented sampling to provide the first basic insights into the suitability of the COI fragment as a species identifying barcode within the centipede genus *Scolopendra*.

## Introduction

The giant centipede *Scolopendra cingulata* Latreille, 1829 is Europe's largest centipede and the most common species of its genus. It is famous for its voracious habits and painful bite as well as its highly variable, often striking color pattern. *Scolopendra* is the only European myriapod genus that can severely harm (e.g. [1]), and in rare cases cause the death of humans [2], [3]. Components of the potent venom were recently discovered to be of potential medical significance as a pain reliever [4] and as an inhibitor to the proliferation of different cancer types and bacteria [5].

**Figure 1:**
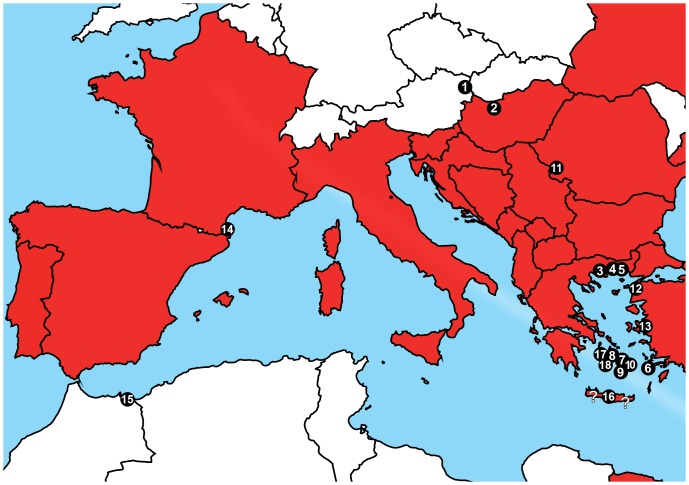
Distribution of *Scolopendra cingulata*. Modified after Lewis [6], showing countries where the species occurs, not exact area of distribution. Numbers correspond to map numbers in Table 1 and question marks represent areas with ambiguous information.

*S. cingulata* is widespread and common surrounding the Mediterranean Sea [6] (Fig. 1), and, in the past, was divided into three geographically distinct clades based on morphology: Western Europe, Italy, and Eastern Europe [7], [8]. While the species, in rare cases, has been dispersed to Central Europe through commerce (e.g., a specimen found in the city of Cologne, Germany; [9]), the natural distribution of *S. cingulata* reaches its northern limit in Romania, Hungary and Austria, where it occurs in small, isolated populations (e. g. [10]). Multiple recent records exist from Hungary [11], [12] where it is listed as an endangered species and receives special protection. In comparison, the Austrian *Scolopendra cingulata* is all but forgotten, not even listed in recent species distribution maps [6], despite the fact that its isolated populations might be at least as endangered and localized as the populations in Hungary.

### *Scolopendra cingulata* in Austria

*Scolopendra cingulata* was mentioned as belonging to the Austrian fauna by Latzel in 1880 [13]. His records, however, refer to the Austro-Hungarian monarchy and these localities now lie in Croatia and Hungary.

The first reference to the species occurring in the Lake Neusiedl area in modern Austria was made by Attems in 1930 [10]. He believed that the species “penetrates the Balkans up to Romania, southern Hungary, and advanced through western Hungary to the Leitha Mountains at the Lake of Neusiedl”. This theory was later refined by Franz [14], [15], who characterized *S. cingulata* as a typical relict form confined to steppe heathland, and to be found in Central Europe only in the Leitha Mts. close to the northern bank of Lake Neusiedl. Szalay [16] argued against the proposed relict scenario and claimed that further populations connecting the scattered distribution would be found in future surveys. Next, Würmli [17] cited these eastern, south-exposed slopes of the Leitha Mts. as the northernmost reliable locality, but added a second even more northern locality. However, the second locality, viz. “Klosterneuburger Au” in Lower Austria, was added with a question mark and without providing any actual specimen records. The latest locality mentioned for the species within modern Austrian borders is an isolated hill called Hackelsberg, which is located between the Leitha Mts. and Lake Neusiedl. This locality is mentioned as the only occurrence of *S. cingulata* in Austria by Kasy in 1979 [18] and is again mentioned by Haider in 2008 [19], both, again, without any specimen records. The old locality Zeiler Berg was mentioned again as an extant locality by Ziegler *et al.* [20] and referred to repeated findings of this centipede from 1981 onwards, made by an annual student field excursion to this region by the ZFMK (see below) headed by one of us (WB). The most recent reference is the exhaustive monograph on Austria's endemic plant and animal species compiled by Rabitsch & Essl in 2009 [21]. Here, *S. cingulata* is not listed in the paragraph on chilopods because it is not believed to be an Austrian endemic or “subendemic”; next to 3 or 4 *Cryptops* species *S. cingulata* is only briefly mentioned as one of a few widely distributed Scolopendromorpha, “at only one single site in northern Burgenland” [22]. Generally, the Austrian population was forgotten or believed to be extinct. In the time since the respective original publications, no recent reports have confirmed the existence of the species at the afore mentioned localities and it is, as already mentioned, exempt from the most current revision of the distribution of old world *Scolopendra* species [6].

Despite the fact that *S. cingulata* represents one of the iconic European myriapod species with a wealth of studies of its ecology (e.g. [23], [24]), morphology (e.g. [8], [25], [26]), behavior (e.g. [3], [27], [28]), and distribution (e.g. [6], [29]), so far molecular studies have only focused on the Greek island populations [30]. In these studies the phylogeography of the species in the Aegean Sea could be reconstructed using a molecular phylogeny of different island populations of *S. cingulata*. Here, we widen the scope by clarifying the evolutionary history of the rediscovered, strongly localized, and potentially endangered population of *S. cingulata* in Austria based on a molecular phylogeny, comparing samples from Austria, Hungary, and Romania, including the first *S. cingulata* samples from the European mainland examined to date. We aim to test the speciation by distance hypothesis, stated by previous authors [10], [14], [15], that *S. cingulata* reached its current relic area in Austria via the Carpathians, through Romania and Hungary, by testing for a correlation between genetic and geographic distance between the different populations. Furthermore, we take advantage of the broad intra-specific sampling to gain first basic insights into the applicability of the COI and 16S fragments as species-specific barcodes inside the genus *Scolopendra*.

## Material and Methods

### Taxon sampling

#### Austria

Specimens of the Austrian *S. cingulata* population were annually watched, studied, and occasionally collected by one of us (WB) at the single known site between 1981–2010, with a handful of specimens stored over the years as vouchers in the collections of the ZFMK. Permits for field studies and specimen collection were granted by the local authorities (Amt der Burgenländischen Landesregierung, Abt. 5 – Anlagenrecht, Umweltschutz und Verkehr). In 2010 it was discovered (by WB & TW) that the specimens from the Austrian population represent the only records of Austrian *Scolopendra* in the last 80 years or more, and its origin became the focus of research interests. To infer the evolutionary history of the Austrian *S. cingulata*, genetic material was collected from specimens from the population in 2011. To limit the impact on the presumably small population, single legs were removed from seven adult specimens, which were released alive. Legs of two additional adults, as well as an already dead specimen, were collected from the same population in 2012.

#### Europe

Since no referenced sequences of *S. cingulata* from the European mainland are available in GenBank (but>70 from Greek islands [30]), additional specimens were analyzed. Because of the supposed eastern origin of the Austrian populations, three Hungarian specimens (Permit: Environmental Conservation Fund No. 027798/2001) and old Museum samples (ZFMK) from Romania, the Greek mainland, and Turkey were added (see Table 1). To rule-out a Western origin of the Austrian population, a sample from SW France was also included. Museum specimens from Italy yielded no suitable DNA. Sequences of each of the main Aegean groups (C1, C2, C3, see [30]) were added from GenBank (Accession numbers: see Table 1).

**Table 1:** Overview of samples included in the present study, with numbers corresponding to the map (Fig. 1), voucher numbers, locality information and accession numbers.

						Accession numbers	
#	Map	Sample ID	Voucher ZFMK #	Species	Locality	COI	16S
1	1	Austria-1 (1)	ZFMK-Sco-1	*S. cingulata*	Austria, Brugenland, Leitha Mts.	KJ812067	KJ812046
2	1	Austria-2 (2)	ZFMK-Sco-2	*S. cingulata*	Austria, Brugenland, Leitha Mts.	KJ812068	KJ812047
3	1	Austria-3 (3)	ZFMK-Sco-3	*S. cingulata*	Austria, Brugenland, Leitha Mts.	KJ812069	n/a
4	1	Austria-4 (4)	ZFMK-Sco-4	*S. cingulata*	Austria, Brugenland, Leitha Mts.	KJ812070	KJ812048
5	1	Austria-5 (5)	ZFMK-Sco-5	*S. cingulata*	Austria, Brugenland, Leitha Mts.	KJ812071	KJ812049
6	1	Austria-6 (6)	ZFMK-Sco-6	*S. cingulata*	Austria, Brugenland, Leitha Mts.	KJ812072	KJ812050
7	1	Austria-7 (7)	ZFMK-Sco-7	*S. cingulata*	Austria, Brugenland, Leitha Mts.	KJ812073	KJ812051
8	1	Austria-8 (8)	ZFMK-Sco-8	*S. cingulata*	Austria, Brugenland, Leitha Mts.	KJ812074	KJ812052
9	1	Austria-9 (9)	ZFMK-Sco-9	*S. cingulata*	Austria, Brugenland, Leitha Mts.	KJ812075	KJ812053
10	1	Austria-10 (10)	ZFMK-Sco-10	*S. cingulata*	Austria, Brugenland, Leitha Mts.	KJ812076	KJ812054
11	1	Austria-11 (11)	Myr 01591	*S. cingulata*	Austria, Brugenland, Leitha Mts.	n/a	KJ812055
12	1	Austria-12 (12)	Myr 01592	*S. cingulata*	Austria, Brugenland, Leitha Mts.	n/a	KJ812056
13	2	Hungary-1 (13)	Myr 01559	*S. cingulata*	Hungary, Vértes Mts., Csákberény, Bucka	KJ812077	KJ812057
14	2	Hungary-2 (14)	Myr 01560	*S. cingulata*	Hungary, Vértes Mts., Csákberény, Bucka	KJ812078	KJ812058
15	2	Hungary-3 (15)	Myr 01561	*S. cingulata*	Hungary, Vértes Mts., Csákvár, Szólókő	KJ812079	KJ812059
16	3	Greece_Kavala-1 (16)	ZFMK-Sco-14	*S. cingulata*	Greece, Kavala	KJ812080	KJ812060
17	3	Greece_Kavala-2 (17)	Myr 00585	*S. cingulata*	Greece, Kavala	KJ812081	n/a
18	4	Greece_Port-Lagos-1 (18)	ZFMK-Sco-13	*S. cingulata*	Greece, Nestos Delta, Port Lagos	KJ812082	KJ812061
19	5	Greece_Port-Lagos-2 (19)	ZFMK-Sco-15	*S. cingulata*	Greece, Nestos Delta, Port Lagos	KJ812083	KJ812062
20	6	*Greece_Nisyros (20)	n/a	*S. cingulata*	Greece, Nisyros	JN688371	JN688421
21	7	*Greece_Koufonisi (21)	n/a	*S. cingulata*	Greece, Koufonisi	JN688365	JN688413
22	8	*Greece_Paros (22)	n/a	*S. cingulata*	Greece, Paros	JN688377	JN688427
23	9	*Greece_Anafi (23)	n/a	*S. cingulata*	Greece, Anafi	JN688350	JN688398
24	10	*Greece_Amorgos (24)	n/a	*S. cingulata*	Greece, Amorgos	JN688349	JN688397
25	11	Romania (25)	ZFMK-Sco-11	*S. cingulata*	Romania, Anina	KJ812086	KJ812065
26	12	Turkey_Troy (26)	ZFMK-Sco-12	*S. cingulata*	Turkey, Troy	KJ812084	KJ812063
27	13	Turkey_Izmir (27)	Myr 00583	*S. cingulata*	Turkey, Izmir	KJ812085	n/a
28	14	France (28)	Myr 01593	*S. cingulata*	France, Banyuls-sur-mer	KJ812087	KJ812064
29	15	oraniensis (29)	Myr 00568	*S. oraniensis*	Marokko, Prov. Nador, Atlas Mts.	KJ812088	KJ812066
30	16	cretica (30)	n/a	*S. cretica*	Greece, Crete	JN688393	JN688440
31	17	*canidens (31)	n/a	*S. canidens*	Greece, Serifos	JN688394	JN688441
32	18	*canidens (32)	n/a	*S. canidens*	Greece, Sifnos	JN688442	n/a

Sequences downloaded from GenBank are marked with an asterisk.

#### Outgroups

Sequences from *S. cretica* Lucas, 1853 and *S. canidens* Newport, 1844 were added from GenBank (Accession numbers: see Table 1). Because no sequences are available on GenBank, a Museum specimen (ZFMK) of *S. oraniensis* Lucas, 1846 from Morocco was also added to the analysis (Table 1).

The total dataset included 30 terminals for the COI (21 newly added), 28 for the 16S (22 newly added), and 30 for the combined dataset (22 newly added), respectively. Locality data (Table 1 and fig. 1) is only given imprecisely because *S. cingulata* is actively traded in the exotic pet market, and the continuous existence of small fringe populations could be harmed by overzealous collectors.

### DNA extraction, amplification, and sequencing

Total genomic DNA was extracted using the DNeasy Blood & Tissue Kit (Qiagen; Valencia, CA, USA).

To study the evolutionary history of the Austrian *S. cingulata* population, fragments of the mitochondrial cytochrome *c* oxidase subunit I (COI) encoding gene and the mitochondrial 16S rRNA (16S) encoding gene were amplified. Both gene fragments have previously been successfully applied to study centipede evolution at both the genus (e.g. [31]–[33]) and species-level (e.g. [30]). Since the 16S fragment only provided low resolution, we decided not to include slower evolving nuclear rDNA genes in this study.

The 16S fragment was amplified using the 16Sa/16Sb primer pair [34]. The COI fragment was amplified from samples Sco01-10 (Austria) using the primer pair LCO1490/HCO2198 [35]. For the remaining samples Nancy [36] was used as an alternative reverse primer. Attempts were also made to amplify the region with the HCOoutout primer [37], [38], but no results of sufficient quality could be obtained even though a wide range of PCR-programs were applied. All polymerase chain reactions (PCR) were carried out using the QIAGEN Multiplex PCR Kit and a T3000 Thermocycler (Biometra). All PCR setups included a positive and negative control. Detailed descriptions of temperature profiles and PCR-mixtures can be found in a previous study [39]. The PCR products were inspected on a 1.5% agarose gel and purified using the QIAquick PCR Purification Kit (Qiagen, following the kit protocol, Valencia, CA, USA). Both strands were sequenced by Macrogen (Macrogen Europe Laboratory, Amsterdam, The Netherlands), using the PCR primers. Sequencing reads were assembled and edited using Geneious 6.0.6 (Biomatters) and Seqman II (DNASTAR, Inc.). Sequence identities were confirmed with BLAST searches [40]. All new sequences were deposited in GenBank (see Table 1 for accession numbers).

### Alignment

All sequences were aligned using the MUSCLE algorithm [41] under the default settings as implemented in Geneious (Biomatters) and edited by hand. Missing ends were filled with N's. The following sites were deleted from the 16S dataset prior to analysis to remove regions of ambiguous homology, mostly regarding the outgroups: 487, 468, 351–353, 345–346, 332–336, 348, 149–150, 20, 1–7. The final alignments consisted of 508 bp (16S), 647 bp (COI) and 1155 bp in the combined dataset. Fasta files of all alignments and tables containing the uncorrected p-distances for both genes can be found in the supplementary material (Tables S1, S2, and Alignments S1, S2, S3).

### Sequence analysis

In all maximum likelihood analyses the dataset was analyzed using the model suggested by the Bayesian Information Criterion (BIC), which was computed by the model test implemented in MEGA 5.1 [42]. The models with the highest fit were HKY+G [43] for the 16S dataset (BIC = 3107.8) and GTR+G [44] for the COI (BIC = 4855.9) and the combined dataset (BIC = 11724.8).

In order to assess the phylogenetic information in our 16S and COI datasets, a likelihood mapping [45] was conducted with TREE-PUZZLE 5.2 [46].

### Maximum Likelihood phylogenetic analysis

**Figure 2:**
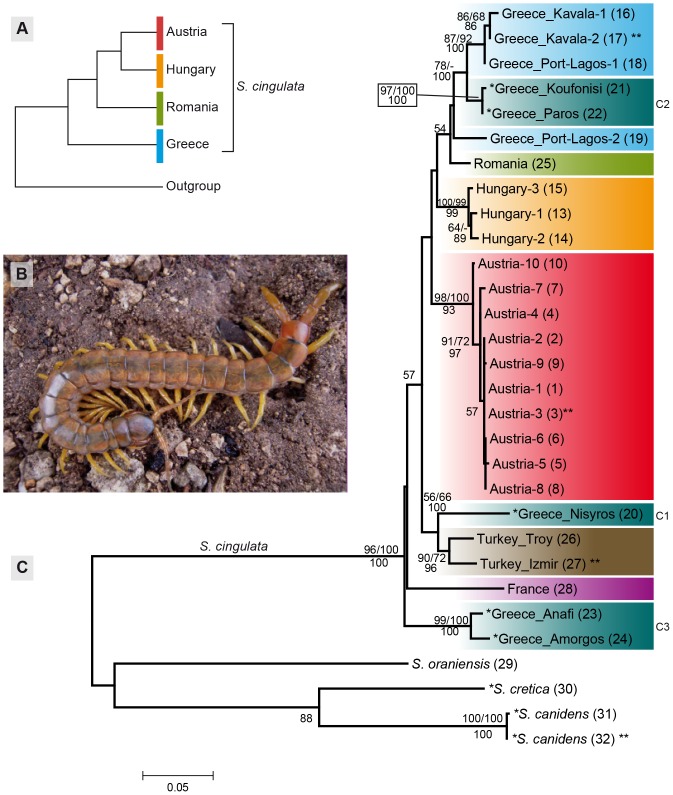
Hypothetical relationships of the northern *Scolopendra cinuglata* and phylogenetic tree recovered in maximum likelihood analysis. **A:** The hypothetical relationships of the northern populations as previously stated by Attems [10] and Franz [14], [15]. **B:** Adult *Scolopendra cingulata* specimen from the Austrian population *in situ*. Photo by Dr. Wolfram Freund. **C:** Maximum likelihood tree of the combined COI and 16S dataset. Numbers represent nodal support values from the maximum likelihood (1000 bootstrap replicates), maximum parsimony analysis (1000 bootstrap replicates) and posterior probabilities from the Bayesian inference (ML/MP/BI). Sequences from GenBank marked with single asterisk in front of name. Samples with two asterisks after name include only the COI sequence. Numbers in parenthesis correspond to sample numbers in Table 1.

All maximum likelihood (ML) analyses were conducted in Mega 5.1 [42]. The initial trees were made by Neighbor joining [47], the heuristic search was conducted with the Nearest Neighbor Interchange algorithm [48] and nodal support values were assessed with 1000 bootstrap pseudoreplicates. The tree obtained by the maximum likelihood analysis of the combined alignments was used for all further discussion and interpretation of the results (Fig. 2C).

### Maximum Parsimony phylogenetic analysis

All Maximum Parsimony (MP) analyses were performed in PAUP* 4.0b10 [49] using the TBR algorithm. Starting trees were obtained via stepwise addition and nodal support was estimated with 1000 bootstrap replicates (unlimited number of trees kept at each replicate). The combined dataset included a total of 304 (16S: 124, COI: 180) parsimony informative characters. For the 16S dataset, 1676 shortest trees with 289 steps were found. For the COI dataset, 175714 shortest trees with 568 steps were found. The analysis of the combined dataset resulted in 2101 shortest trees with 867 steps. Strict consensus trees were produced for all datasets (trees not shown). Nodal support values of the MP bootstrap analysis are displayed in Figure 2C.

### Bayesian phylogenetic analysis

Bayesian inference (BI) was conducted using MrBayes 3.1.2 [50]. Each dataset was analyzed with the model suggested by the model test implemented in MEGA 5.1 [42], as described above. The combined dataset was partitioned to allow unlinked models for the two genes. The model parameters (priors) were left unfixed to allow estimation from the dataset, as suggested by the MrBayes manual. The analysis was performed using 3,000,000 Monte Carlo Markov Chain (MCMC) generations of three hot and one cold chain in two parallel runs, sampling trees every 100th generation. The likelihood values for the parallel runs were inspected manually and the generations prior to a stable value were discarded as burn-in. The burn-in was set to 3600, 3100 and 8400 generations for the COI, 16S and concatenated dataset, respectively. Nodal support values are displayed in Figure 2C.

### Analysis of the Evolutionary History of the Austrian *S. cingulata* population

In order to further test the hypothesis of a speciation by distance scenario, as stated by previous authors [10], [14], [15], we compared the genetic and geographic distances between the Austrian *S. cingulata* and those from France, Greece, Turkey, Romania and Hungary.

The *S. cingulata* COI sequences and geographic distances were pooled according to populations (Tab. 2) and Kendall's Tau correlation test [51] was performed, as the data was unsuitable for a Mantel test [52]. Kendall's Tau allows to test for a correlation between two variables where the measurements are not equidistant and the data is non-parametric. To assess whether the data are uncorrelated or not, the two-tailed probability test was also performed. All tests were performed in PAST [53].

**Table 2:** Geographic and genetic distances (COI, uncorrected p) between the Austrian (Map #1) and all other populations.

Map #	Localities	Distance [km]	Distance COI [%]
2	Hungary, Vértes Mts.	138	4,4
3	Greece, Kavala	989	2,4
4	Greece, Port Lagos I	1016	2,3
5	Greece, Port Lagos II	1016	2,1
6	Greece, Nisyros	1526	6,0
7	Greece, Koufonisi	1422	2,5
8	Greece, Paros	1395	2,5
9	Greece, Anafi	1488	5,8
10	Greece, Amorgos	1444	6,0
11	Romania	504	2,9
12	Turkey, Troy	1151	3,5
13	Turkey, Izmir	1353	3,1
14	France	1228	5,8

### Barcode evaluation

To provide preliminary insights into the suitability of the COI fragment as a species-delimiting barcode, the frequency distribution of all pairwise uncorrected p-distances were analyzed. If the COI fragment is suitable for species identification within *Scolopendra*, a (barcode) gap should exist between the inter- and intra-specific distances [54], [55].

## Results

### Sequence Data

The sequencing was successful for most specimens, with the exception of two (one from Izmir, Turkey; and one from Kavala, Greece) of which only the COI was obtained, and of two specimens from Austria of which only the 16S sequence could be obtained.

In the COI dataset, the A, T, C and G frequencies were 0.35, 0.27, 0.22 and 0.17, and in the 16S dataset, they were 0.30, 0.39, 0.09 and 0.22, respectively. The sequence composition in our COI dataset shows a clear bias towards A and T, which has been shown to be common within chilopods [56] and arthropods in general [57]–[60].

**Figure 3:**
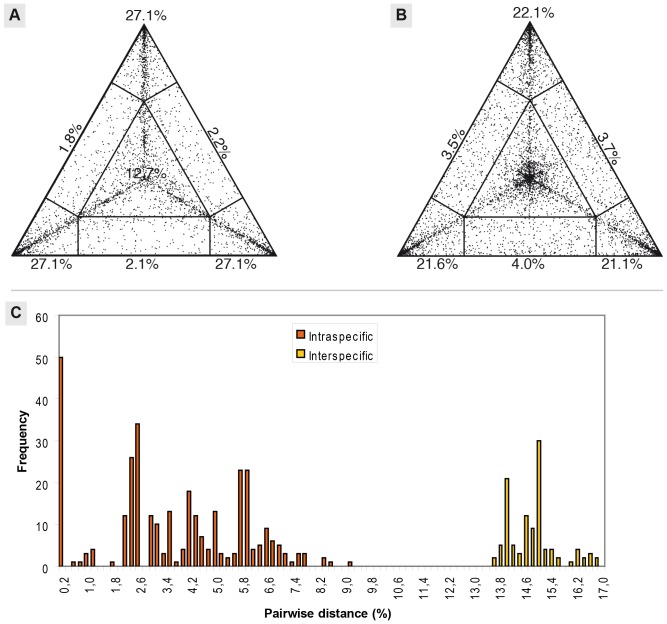
Results from likelihood mapping and barcode-gap analyses. **A:** Likelihood Mapping for COI dataset. **B:** Likelihood mapping for the 16S dataset. **C:** Barcode-gap analysis: Frequency distribution of the pairwise uncorrected p-distances of the COI sequences. Orange bars show intraspecific distances and yellow bars represent interspecific distances.

The likelihood-mapping showed a higher amount of phylogenetic information content in the COI dataset than in the 16S (Figs. 3A, B). The 16S analysis resulted in 24.0% unresolved trees and a total of 11.2% partially resolved trees (Fig. 3B). The COI analysis, on the other hand, resulted in only 12.7% unresolved trees and a total of 6.1% partially resolved trees (Fig. 3A).

### Molecular Phylogenetic Analyses

The trees obtained by the maximum likelihood analysis of the combined dataset, with the added support values of the MP and Bayesian analyses, are utilized for the presentation of results (Fig. 2C).

In the analysis of the combined dataset, the *S. cingulata* samples form a well-supported (ML = 96%, MP = 100%, PP = 100%) clade against the out-group (*S. cretica*, *S. canidens*, *S. oraniensis*) (Fig. 2C). The genetic distances between *S. cingulata* and its three congeners are high (COI: 13.5–16.8%, 16S: 19.3–23.0%). In the out-group, *S. oraniensis* branches off basally, where *S. cretica* and *S. canidens* form a group (88% ML support). *S. oraniensis* seems to be only slightly more closely related to *S. canidens* (uncorr. p-dist.: COI: 14.5%, 16S: 22.7%), than to *S. cretica* (uncorr. p-dist.: COI: 15.1%, 16S: 20.9%).

**Figure 4:**
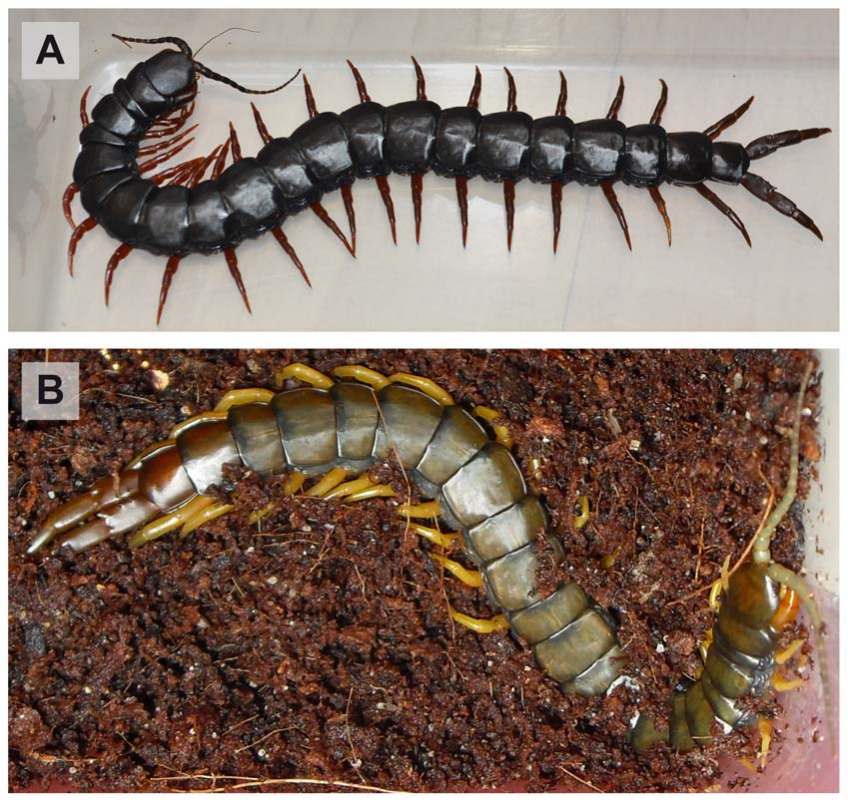
Sympatrical *Scolopendra cingulata* color morphs from Port Lagos (Greece), *ex-situ*. **A:** Red legged morph with black body. **B:** Yellow legged morph with green-brown body.

Within *S. cingulata* the basal-most branch consists of a well-supported group (99/100/100) containing two Greek island specimens, representing group C3 of previous analyses [30]. The next split in the tree places the western European specimen outside the only weakly supported (57% ML), clade of the remaining samples (Fig. 2C). Within the clade, the sample from Nisyros (C1, [30]) stands basally in a well-supported (56/66/100) group with the two specimens from Turkey. The sister-group to the Greek-Turkish clade is poorly supported. Inside the latter, the Austrian *S. cingulata* represent the basal-most group. The group's monophyly receives strong support (98/100/93) and it contains only a single haplotype in both the COI and 16S gene. The sister-group of the Austrian *S. cingulata* is a clade consisting of specimens from Hungary, Romania and Greece (Fig. 2C). Basally, the Hungarian specimens form a well-supported monophyletic clade (100/99/99), while their sister-groups are less well supported. The three Hungarian samples, from localities less than 1 km apart, display different COI haplotypes with small genetic distances (COI: 0.6–1.0%, 16S: 0.0%). The first weakly supported (57/-/-) split within the sister-group to the Hungarian samples, places the Romanian sample outside of a clade containing the remaining Greek samples. Within the Greek samples (excluding the basal Greek Island C3 and the Turkish-Nisyros C1), three well-supported clades can be distinguished: (1) one specimen (red legged, Fig. 4A) from Port Lagos, which forms the sister-group to (2) the island samples C2 (97/100/100), and (3) the well-supported (87/92/100) Greek mainland specimens from Kavala and Port Lagos (yellow legged, Fig. 4B).

### Evolutionary History of the Austrian *S. cingulata* population

The speciation by distance scenario, as suggested by Attems [10] and Franz [14], [15], postulates that *S. cingulata* could have reached Austria from an eastern refugium or point of origin via Hungary and Romania. A positive correlation between the geographic and genetic distance, as would be expected under said scenario, could not be proven. Although a weak positive correlation was detected (tau = 0.34213), the probability test failed to support this scenario (p = 0.1035).

### Barcode evaluation

A clear gap was found between the intra- and interspecific distances (Fig. 3C). The average intraspecific distance was 6.4% and the highest was 9.1% between the single French specimen and one specimen from the Greek island Nisyros. The average interspecific distance was 14.8% and the lowest was 13.5% between one of the Hungarian *S. cingulata* specimens and *S. cretica*, as well as between *S. cretica* and the *S. canidens* specimen from Sifnos.

## Discussion

### Sequence Data – Barcode evaluation

Our analysis of the COI dataset regarding the suitability of the sequence as a species-delimiting barcode showed a clear “barcode gap” between the intra- and interspecific distances. Such a gap was also reported for the North-African representatives of the lithobiid genus *Eupolybothrus*, with the lowest interspecific distance of 16.61% and intraspecific distances of 1.4% and 0.3% [33]. However, a finer geographical sampling of all taxa would be necessary to validate our findings as high intraspecific distances have been reported for the New Caledonian endemic species *Cryptops pictus*, with a divergence of up to 23.8% [32], as well as several instances of overlapping inter- and intraspecific distances in Bavarian Chilopoda [56]. The latter was suggested to be due to possible cryptic species or alternatively long separated haplotypes, which would concur with the findings of Wiemers & Fidler [61]. They showed that barcode gaps could be artefacts resulting from incomplete geographical sampling of widespread species. Using the 16S fragment might pose a better option, as the highest intraspecific divergence within our *S. cingulata* samples was only 4.5%, much lower than in the COI (9.1%), and the lowest interspecific divergence was still at 10.2%, similar to the one of the COI dataset (13.5%), between the closely related *S. cretica* and *S. canidens*, and much higher between the other species (19.3–23.0%). The 16S fragment would likely increase the accuracy of the barcode, but at the cost of the population genetic insight.

### Evolutionary History of the Austrian *S. cingulata*

The expected hypothetical phylogenetic tree (Fig. 2A), according to the evolutionary hypothesis by past authors [10], [14], [15], could not be recovered in any of the conducted analyses of the mitochondrial genes (Fig. 2C). Although the most interesting splits received weak statistical support in our phylogenetic analyses, it remains clear that the Austrian, Hungarian, Romanian, and northern Greek specimens are closely related. The study by Simaiakis *et al.* [30] recovered the split between the eastern Aegean Islands (C1) and the northern and central Cyclades (C2) to most likely have happened approximately 10.12 Mya. In our analysis of the combined COI and 16S datasets, the Austrian population is the first to split from the branch leading to the representatives of the C1 group (Fig. 2C). This might indicate that the lineage of the Austrian population was established much earlier than the possible repopulation of the Carpathian Basin following the last glacial period. Furthermore, the fact that both of the postulated closest relatives to the Austrian *Scolopendra* population, the Hungarian and Romanian specimens, have a lower genetic distance to the northern Greek samples (Port Lagos) than to each other or to the Austrian population indicates that the underlying pattern is more intricate than what can be explained by a single range expansion of *S. cingulata* population founders out of a Mediterranean refugium.

Our study failed to significantly prove the postulated speciation by distance scenario suggested by Attems [10]. This implies that more complex mechanisms and/or events preceding the last glacial period could have shaped the northernmost distribution of *Scolopendra cingulata* in Europe. Multiple independent recolonizations would correspond with the view of Varga [62], who concludes that populations from multiple small meso- and microclimatically favourable sites at the fluctuating borderlines of the Mediterranean refugial and periglacial belts played a significant part in the postglacial repopulation of the Carpathian Basin in several insect groups. Though this scenario of multiple recolonizations seems probable, further and denser sampling, especially of geographically close western populations and the populations in the regions surrounding the former periglacial belts, would be necessary to confirm our theory, as the current sampling does not provide the resolution required to draw any firm conclusions.

### A valuable relict in a microrefugium: The last habitat of the Austrian *S. cingulata*

Franz [14], [15] proposed that the Austrian and Hungarian *S. cingulata* populations are relicts of a previous wider distribution during the post-glacial climatic optimum, which later became isolated because of the following cooler climate and the expansion of the forest. Szalay [16] on the other hand expected further populations to be found, even connecting the populations to the main area of distribution. This does not seem to be the case, as such connections still have not been found after numerous excursions to the area over the span of 30 years. Furthermore, many of the cited localities in the literature are, at best, implausible. The locality Klosterneuburger Au in Lower Austria, mentioned by Würmli [17] seems absolutely unlikely to be a suitable habitat for this thermophilous centipede, as “Au” means the gallery forest along the Danube River close to the city of Klosterneuburg. The locality at the Hackelsberg, as mentioned by Haider [19], is also dubious. The accompanying photograph shows a specimen from a doubtlessly Mediterranean rather than Austrian population; it is actually from southern France as communicated to us by the photographer, F. Geller-Grimm. All of the above mentioned accounts are lacking specimen records, and can therefore not be validated. Therefore, the Austrian, and to some extent the Hungarian, *Scolopendra cingulata* populations should be viewed as biogeographical relicts (sensu Lomolino *et al.* [63]).

The Austrian population, despite its low genetic variation, represents a completely unique haplotype within the species (Splitstree analysis: Data not presented). It has been shown that peripheral relict populations of widespread species can harbor unique genetic information [64] and that adaptations, which were gained during the range expansions, are lost when the range becomes restricted [65]. Additionally, relict populations might also be important during future range expansions or shifts, enabling *S. cingulata* to colonize a large area faster than what would be possible through diffusional migration along a single expanding front [66]. Thus, even though *S. cingulata* shows a widespread distribution on a continental scale, the Austrian population, which is a significant part of the genetic diversity, could be important to the future survival of the species and should therefore be protected.

The small area inhabited by the Austrian population, an exposed southern slope with scattered boulders of varying sizes, should probably be considered a microrefugium, which can be defined as a small area with local favorable environmental features in which small populations can survive outside their main distribution area, protected from the unfavorable regional environmental conditions [67], [68]. Favorable microclimatical conditions and the neglect by farmers have probably allowed the isolated population of the species to survive. This is supported by the syntopical occurrence of protected thermophilous vertebrates (e.g. *Lacerta viridis, Zamenis longissimus*) and several thermophilous insects, including the rare ground beetle *Carabus hungaricus*, *Mantis religiosa*, *Platycleis grisea*, and, historically, *Sago pedo*. It is also likely that the unique habitat presently hosts further thermophilous taxa. Even if the climatic conditions should change, the site is still likely to retain a special microclimate relative to the surroundings and will therefore possibly remain as a microrefugium for a new set of species [66]. Consequently, the habitat is not only worthy of protection to secure the current *S. cingulata* population, but also to protect further species now and in the future.

### Study of the European mainland *S. cingulata* populations versus other *S. cingulata* studies

Minelli [7] proposed that *S. cingulata* populated southern Europe (Iberian-, Italian and the Balkan Peninsula) quite recently. In contrast, a later study based on distributional patterns [29] suggested that the species differentiation in the Mediterranean Basin happened less than 5.5 Mya, or, alternatively, between 9 and 12 Mya via either the Balkans or northern Africa. A recent study based on molecular data supports the latter view, suggesting the time of divergence for two main lineages of Aegean *S. cingulata* to have been approximately 10.5 Mya years ago [30]. However, our data suggests that the Aegean islands were most likely colonized from multiple directions as supported by the fact that the representatives from the C2 group cluster within the Greek mainland samples and that the C1 sample clusters with the Turkish mainland samples (Fig. 2C). This could imply that the formation of the mid-Aegean trench might be irrelevant to the C1/C2 split, rendering the calibration of the phylogeny in the previous analysis [30] inaccurate.

An additional study of *S. cingulata* populations based on morphometric data revealed an east-west gradient within the species [8], suggesting a colonization of central and southern Europe from the easternmost parts of its range (Asia Minor and Middle East) via the Balkans and Northern Africa. Our analyses support the notion of separate colonization events, because the French sample is recovered in a basal position relative to the eastern European samples. If the French population had originated via the Balkans, a closer relationship with the Austrian or Greek populations would have been expected. However, given the limitations of the current sampling, our results are concurring with but not corroborating the recently proposed multiple colonizations of the European continent [8]. Regrettably, the samples used for the present study were for the most part juveniles, prohibiting a morphometric evaluation.

### Two sympatric, unrelated different color morphs in Greek *S. cingulata*

The two Greek mainland samples from Port Lagos were animals of two different color morphs; one had red legs and a black body (Fig. 4A) while the other had yellow legs with a green-brown body (Fig. 4B). Such extreme color variation is not rare in *Scolopendra* species. For example, Shelley [69] reports that *S. viridis* in North America ranges from a solid green to yellow in variations with longitudinal or transverse stripes. However, the genetic basis of these variations is completely unknown. It is especially interesting that the two sympatrically occurring morphs do not seem to be each other's closest relatives. The gray animal clusters with the other Greek mainland samples from Kavala, which were brown with yellow legs, and the red-black animal is resolved as the sister taxon to the group containing the Greek mainland samples and the samples from the Greek islands Paros and Koufonisi (Fig. 2C). Further sampling would be required to confirm if there is any phylogenetic information behind the different morphs or if the variation is also present within one lineage. Presently, we can only conclude that the differentiation is clearly within the intra-specific range, since the divergence between the two samples is only 2.3% (COI).

### Analysis problems

The likelihood-mapping analyses we conducted show that the 16S fragment did not provide much phylogenetic information on the intraspecific level, as a large portion of the trees remained completely or at least partially unresolved. This is also evident in the trees produced by our phylogenetic analyses, where the dataset only provides some resolution in the most basal splits. The lack of intraspecific variation (highest divergence of 4.3%) prompted us to omit the gene from the analysis of the evolutionary history (but not the phylogeny, see Fig. 2C). A faster evolving gene than COI or 16S is needed to further elucidate the evolutionary history of the northernmost *S. cingulata* and for future studies in the genus *Scolopendra* at the species level. While fragments of the 12S [30], [70] and 28S gene [70]–[73] have been employed in previous centipede studies, these genes seem to be even slower evolving than 16S. ITS (internal transcribed spacer) might be a future alternative, but it does not seem to provide more species level information than the COI-fragment [32].

Unfortunately, a microsatellite study – often the method of choice [74] for population genetic studies in insects (e.g. [75]) and vertebrates (e.g. [76]) – has never been conducted in Chilopoda. A cheaper and easier alternative might be using AFLPs since the method does not require specific primers or any previous knowledge about the sequences [74], [77]. However, an AFLP study is not possible with old museum specimens. As previously mentioned, a more fine-tuned and denser sampling across the whole distributional range would also vastly improve the conclusiveness of our analyses.

### Outlook

To test the hypothesis of multiple independent colonization events and elucidate the phylogeography of the northernmost populations of *Scolopendra cingulata* further, a finer geographic taxon sampling as well as the application of other molecular markers, as discussed above, is absolutely essential. Including further peripheral populations will be difficult, as they are extremely scattered and often restricted to very small areas. Within Asturia *S. cingulata* is only known from the sample locality (which is <1000 m^2^) and the distribution in Hungary was only revised recently [11], [12], [78], so that precise localities are available. Such extremely localized fringe populations are difficult to localize and sample. However, including further samples from the main distribution area would be very interesting, since several thermophilous taxa in the Carpathian basin show connections to the Balkans, southern Russia and Asia Minor [79].

## Supporting Information

Table S1 **Uncorrected p-distances of 16S alignment.** Computed with Mega5 [42].
(XLS)

Table S2 **Uncorrected p-distances of COI alignment.** Computed with Mega5 [42].
(XLS)

Alignment S1 **Muscle [41]** **alignment of** ***S. cingulata*** **16S sequences.**
(TXT)

Alignment S2 **Muscle [41]** **alignment of** ***S. cingulata*** **COI** ***sequences*** **.**
(TXT)

Alignment S3 **Muscle [41]** **alignment of concatenated** ***S. cingulata*** **16S and COI sequences.**
(TXT)

## References

[1] Yao X, Dong Q, Chen Y, Feng Z, Li Y (2013) Acute disseminated encephalomyelitis following biting by a scolopendra subspinipes mutilans. Clin Toxicol (Phila) 51: 519–520 10.3109/15563650.2013.804929 23731374

[2] Serinken M, Erdur B, Sener S, Kabay B, Cevik A (2005) A case of mortal necrotizing fasciitis of the Trunk resulting from a centipede (Scolopendra moritans) Bite. Internet Journal of Emergency Medicine 2: 1.

[3] Voigtländer K (2011) Chilopoda - Ecology. In: Minelli A, editor. Treatise on Zoology - Anatomy, Taxonomy, Biology, The Myriapoda I. Leiden: Koninklijke Brill. pp. 309–326.

[4] Yang S, Xiao Y, Kang D, Liu J, Li Y, et al (2013) Discovery of a selective NaV1.7 inhibitor from centipede venom with analgesic efficacy exceeding morphine in rodent pain models. Proc Natl Acad Sci U S A 110: 17534–17539 10.1073/pnas.1306285110 24082113 PMC3808613

[5] Kong Y, Hui J, Shao Y, Huang S, Chen H, et al (2013) Cytotoxic and anticoagulant peptide from Scolopendra subspinipes mutilans venom. African J Pharm Pharmacol 7: 2238–2245 10.5897/AJPP2013.3765

[6] Lewis JGE (2010) A key and annotated list of the *Scolopendra* species of the Old World with a reappraisal of *Arthrorhabdus* (Chilopoda: Scolopendromorpha: Scolopendridae). Int J Myriap 3: 83–122 10.1163/187525410X12578602960380

[7] Minelli A (1983) Note critiche sui Chilopodi della Sardegna. Lavori della Società Italiana di Biogeografia Nuova Serie 8: 401–416.

[8] Simaiakis SM, Giokas S, Korsós Z (2011) Morphometric and meristic diversity of the species Scolopendra cingulata Latreille, 1829 (Chilopoda: Scolopendridae) in the Mediterranean region. Zool Anzeiger - A J Comp Zool 250: 67–79 10.1016/j.jcz.2010.11.006

[9] Decker P, Hanning K (2011) Checkliste der Hundert- und Tausendfüßer (Myriapoda: Chilopoda, Diplopoda) Nordrhein-Westfalens. Abhandlungen aus dem Westfälischen Museum für Naturkunde 73: 48.

[10] Attems C (1930) Myriapoda. 2. Scolopendromorpha. Das Tierreich 54: 1–308.

[11] Dányi L (2006) Az öves szkolopendra (Scolopendra cingulata Latreille, 1829) elsõ elõfordulási adatai a Bakonyból és újabban felfedezett élõhelyei a Vértesben. Folia musei historico-naturalis bakonyiensis a bakonyi természettudományi múzeum közleményei 23: 27–31.

[12] Korsós Z, Dányi L, Kontschán J, Murányi D (2006) Az öves szkolopenda (Scolopendra cingulata Latr., 1829) magyarországi állományainak helyzete. Természetvédelmi Közlemények 12: 155–163.

[13] Latzel R (1881) Die Myriopoden der österreichisch-ungarischen Monarchie. Erste Hälfte: Die Chilopoden. Mit 10 lithogr. Tafeln. Wien 1880, Alfred Hölder. 8°. 228 u. XV. Stn. Mitt Mus Naturkunde Berl Dtsch Entomol Z 25: 92 10.1002/mmnd.18810250115

[14] Franz H (1936) Die thermophilen Elemente der mitteleuropäischen Fauna und ihre Beeinflussung durch die Klimaschwankungen der Quartärzeit. Zoogeographica 3: 159–320.

[15] Franz H (1938) Steppenrelikte in Sudöstmitteleuropa und ihre Geschichte. VII. Internationaler Kongress für Entomologie 1: 102–117.

[16] Szalay L (1956) Über die geographische Verbreitung von Scolopendra cingulata Latr. (Chilopoda). Zool Anz 157: 35–36.

[17] Würmli M (1972) Myriapoda, Chilopoda. Catalogus Faunae Austriae, 11a. Springer Verlag, Wien. 1–16.

[18] Kasy F (1979) Die Schmetterlingsfauna des Naturschutzgebietes Hackelsberg, Nordburgenland. Zeitschrift der Arbeitsgemeinschaft Österreichischer Entomologen 30: 1–44.

[19] Haider M (2008) Jungerberg und Hackelsberg. Dokumentation bedeutender Kulturlandschaften in der grenzüberschreitenden Region Neusiedler See. Naturschutzbund Burgenland, Eisenstadt 1–8.

[20] Ziegler T, Vences M, Bohme W (1998) Das Gebiet des Neusiedlersees. Wenig beachtete zoologische Besonderheiten. TI-Magazin 139: 71–74.

[21] Rabitsch W, Essl F (2009) Endemiten – Kostbarkeiten in Österreichs Pflanzen- und Tierwelt. Klagenfurt & Wien: Naturwissenschftlicher Verein für Kärnten und Umweltbundesamt GmbH.

[22] Christian E (2009) Chilopoda (Hundertfüsser). In: Rabitsch W, Essl F, editors. Endemiten – Kostbarkeiten aus Österreichs Pflanzen- und Tierwelt. Klagenfurt & Wien: Naturwissenschaftlicher Verein für Kärnten und Umweltbundesamt GmbH. pp. 542–545.

[23] Kaltsas D, Simaiakis S (2012) Seasonal patterns of activity of Scolopendra cretica and S. cingulata (Chilopoda, Scolopendromorpha) in East Mediterranean maquis ecosystem. Int J Myriap 7: 1 10.3897/ijm.7.2133.

[24] Radl RC (1992) Brood Care in Scolopendra cingulata LATREILLE (Chilopoda: Scolopendromorpha). Berichte des naturwissenschaftlichen-medizinischen Verein Innsbruck 10: 123–127.

[25] Kaufman ZS (1962) The structure of digestive tract in Scolopendra cingulata Latr. (Chilopoda). Zool Zhurnal 41: 859–869.

[26] Chajec L, Sonakowska L, Rost-Roszkowska MM (2014) The fine structure of the midgut epithelium in a centipede, Scolopendra cingulata (Chilopoda, Scolopendridae), with the special emphasis on epithelial regeneration. Arthropod Struct Dev 43: 27–42 10.1016/j.asd.2013.06.002 23831526

[27] Klingel H (1960) Vergleichende Verhaltensbiologie der Chilopoden Scutigera coleoptrata L. (“Spinnenassel”) und Scolopendra cingulata Latreille (Skolopender). Z Tierpsychol 17: 11–30 10.1111/j.1439-0310.1960.tb00191.x

[28] Pontuale G, Romagnoli P, Maroli M (1997) Biology and pathology of Scolopendra cingulata Latreille, 1829 (Chilopoda: Scolopendridae) stings. Ann Ist Super Sanita 33: 241–244. 9470247

[29] Simaiakis S, Mylonas M (2008) The Scolopendra species (Chilopoda: Scolopendromorpha: Scolopendridae) of Greece (E-Mediterranean): a theoretical approach on the effect of geography and palaeogeography on their distribution. Zootaxa 53: 39–53.

[30] Simaiakis S, Dimopoulou A, Mitrakos A, Mylonas M, Parmakelis A (2012) The evolutionary history of the Mediterranean centipede Scolopendra cingulata (Latreille, 1829)(Chilopoda: Scolopendridae) across the Aegean archipelago. Biol J Linn Soc 105: 507–521 10.1111/j.1095-8312.2011.01813.x

[31] Murienne J, Edgecombe GD, Giribet G (2010) Including secondary structure, fossils and molecular dating in the centipede tree of life. Mol Phylogenet Evol 57: 301–313 10.1016/j.ympev.2010.06.022 20601003

[32] Murienne J, Edgecombe GD, Giribet G (2011) Comparative phylogeography of the centipedes *Cryptops pictus* and *C. niuensis* (Chilopoda) in New Caledonia, Fiji and Vanuatu. Org Divers Evol 11: 61–74 10.1007/s13127-011-0041-7

[33] Stoev P, Akkari N, Zapparoli M, Porco D, Enghoff H, et al (2010) The centipede genus *Eupolybothrus* Verhoeff, 1907 (Chilopoda: Lithobiomorpha: Lithobiidae) in North Africa, a cybertaxonomic revision, with a key to all species in the genus and the first use of DNA barcoding for the group. Zookeys 77: 29–77. 10.3897/zookeys.50.504 PMC3088018 21594115

[34] Xiong B, Kocher TD (1991) Comparison of mitochondrial DNA sequences of seven morphospecies of black flies (Diptera: Simuliidae). Genome 34: 306–311 10.1139/g91-050 2055453

[35] Folmer O, Black M, Hoeh W, Lutz R, Vrijenhoek R (1994) DNA primers for amplification of mitochondrial cytochrome c oxidase subunit I from diverse metazoan invertebrates. Mol Mar Biol Biotechnol 3: 294–299. 7881515

[36] Simon C, Frati F, Beckenbach A, Crespi B, Liu H, et al (1994) Evolution, weighting, and phylogenetic utility of mitochondrial gene sequences and a compilation of conserved polymerase chain reaction primers. Ann Entomol Soc Am 87: 651–701.

[37] Prendini L (2005) Comment on “Identifying spiders through DNA barcodes”. Can J Zool 83: 498–504 10.1139/Z05-025

[38] Schwendinger PJ, Giribet G (2005) The systematics of the south-east Asian genus *Fangensis* Rambla (Opiliones: Cyphophthalmi: Stylocellidae). Invertebr Syst 19: 297 10.1071/IS05023

[39] Wesener T, Raupach MJ, Sierwald P (2010) The origins of the giant pill-millipedes from Madagascar (Diplopoda: Sphaerotheriida: Arthrosphaeridae). Mol Phylogenet Evol 57: 1184–1193 10.1016/j.ympev.2010.08.023 20813191

[40] Altschul SF, Madden TL, Schäffer a a, Zhang J, Zhang Z, et al (1997) Gapped BLAST and PSI-BLAST: a new generation of protein database search programs. Nucleic Acids Res 25: 3389–3402. 9254694 10.1093/nar/25.17.3389 PMC146917

[41] Edgar RC (2004) MUSCLE: multiple sequence alignment with high accuracy and high throughput. Nucleic Acids Res 32: 1792–1797 10.1093/nar/gkh340 15034147 PMC390337

[42] Tamura K, Peterson D, Peterson N, Stecher G, Nei M, et al (2011) MEGA5: molecular evolutionary genetics analysis using maximum likelihood, evolutionary distance, and maximum parsimony methods. Mol Biol Evol 28: 2731–2739 10.1093/molbev/msr121 21546353 PMC3203626

[43] Hasegawa M, Kishino H, Yano T (1985) Dating of the human-ape splitting by a molecular clock of mitochondrial DNA. J Mol Evol 22: 160–174. 3934395 10.1007/BF02101694

[44] Nei M, Kumar S (2000) Molecular Evolution and Phylogenetics. New York: Oxford University Press.

[45] Strimmer K, Haeseler AVon (1996) Quartet puzzling: a quartet maximum-likelihood method for reconstructing tree topologies. Mol Biol Evol 964–969.

[46] Schmidt HA, Strimmer K, Vingron M, von Haeseler A (2002) TREE-PUZZLE: maximum likelihood phylogenetic analysis using quartets and parallel computing. Bioinformatics 18: 502–504. 11934758 10.1093/bioinformatics/18.3.502

[47] Saitou N, Nei M (1987) The neighbor-joining method: a new method for reconstructing phylogenetic trees. Mol Biol Evol 4: 406–425. 3447015 10.1093/oxfordjournals.molbev.a040454

[48] Camin J, Sokal R (1965) A method for deducing branching sequences in phylogeny. Evolution 19: 311–326.

[49] Swofford DL (2003) PAUP*–Phylogenetic Analysis Using Parsimony (* and Other Methods), Version 4.0 b10. Sunderland, MA: Sinauer Associate.

[50] Ronquist F, Huelsenbeck JP (2003) MrBayes 3: Bayesian phylogenetic inference under mixed models. Bioinformatics 19: 1572–1574 10.1093/bioinformatics/btg180 12912839

[51] Kendall MG (1938) A new measure of rank correlation. Biometrika 30: 81–93 10.2307/2332226

[52] Mantel N (1967) The detection of disease clustering and a generalized regression approach. Cancer Res 27: 209–220 10.1038/214637b0 6018555

[53] Hammer Ø, Harper DAT, Ryan PD (2001) Past: Paleontological Statistics Software Package for Education and Data Analysis. Palaeontologia electronica 4: 1–9 10.1016/j.bcp.2008.05.025

[54] Hebert PDN, Cywinska A, Ball SL, deWaard JR (2003) Biological identifications through DNA barcodes. Proc Biol Sci 270: 313–321 10.1098/rspb.2002.2218 12614582 PMC1691236

[55] Barrett RDH, Hebert PDN (2005) Identifying spiders through DNA barcodes. Can J Zool 83: 481–491 10.1139/Z05-024

[56] Spelda J, Reip HS, Oliveira-Biener U, Melzer RR (2011) Barcoding Fauna Bavarica: Myriapoda - a contribution to DNA sequence-based identifications of centipedes and millipedes (Chilopoda, Diplopoda). Zookeys 139: 123–139 10.3897/zookeys.156.2176 PMC3253575 22303099

[57] France SC, Kocher TD (1996) Geographic and bathymetric patterns of mitochondrial 16S rRNA sequence divergence among deep-sea amphipods, Eurythenes gryllus. Mar Biol 126: 633–643 10.1007/BF00351330

[58] Held C (2000) Phylogeny and biogeography of serolid isopods (Crustacea, Isopoda, Serolidae) and the use of ribosomal expansion segments in molecular systematics. Mol Phylogenet Evol 15: 165–178 10.1006/mpev.1999.0739 10837149

[59] Wetzer R (2001) Hierarchical analysis of mtDNA variation and the use of mtDNA for isopod (Crustacea: Peracarida: Isopoda) systematics. Contributions to Zoology 70: 23–39.

[60] Wetzer R, Martin JW, Trautwein SE (2003) Phylogenetic relationships within the coral crab genus Carpilius (Brachyura, Xanthoidea, Carpiliidae) and of the Carpiliidae to other xanthoid crab families based on molecular sequence data. Mol Phylogenet Evol 27: 410–421 10.1016/S1055-7903(03)00021-6 12742746

[61] Wiemers M, Fiedler K (2007) Does the DNA barcoding gap exist? - a case study in blue butterflies (Lepidoptera: Lycaenidae). Front Zool 4: 8 10.1186/1742-9994-4-8 17343734 PMC1838910

[62] Varga Z (2010) Extra-Mediterranean Refugia, Post-Glacial Vegetation History and Area Dynamics in Eastern Central Europe. In: Habel JC, Assmann T, editors. Relict Species - Phylogeography and Conservation Biology. Berlin Heidelberg: Springer. pp. 57–59.

[63] Lomolino MV, Riddle RB, Brown JH (2006) Biogeography. Sunderlan, MA: Sinauer.

[64] Cassel-Lundhagen A (2010) Peripheral Relict Populations of Widespread Species; Evolutionary Hotspots or Just More of the Same? In: Habel JC, Assman T, editors. Relict Species - Phylogeography and Conservation Biology. Berlin Heidelberg: Springer. pp. 267–275.

[65] Bennett KD, Tzedakis PC, Willis KJ (1991) Quaternary refugia of north European trees. J Biogeogr 18: 103–115.

[66] Mosblech NAS, Bush MB, van Woesik R (2011) On metapopulations and microrefugia: palaeoecological insights. J Biogeogr 38: 419–429 10.1111/j.1365-2699.2010.02436.x

[67] Rull V, Schubert C, Aravena R (1988) Palynological studies in the Venezuelan Guayana Shield: preliminary results. Curr Res Pleistocene 5: 54–56.

[68] Rull V (2009) Microrefugia. J Biogeogr 36: 481–484 10.1111/j.1365-2699.2008.02023.x

[69] Shelley RM (2002) A synopsis of the North American centipedes of the order Scolopendromorpha (Chilopoda). Virginia Museum of Natural History Memoir 5: 1–108.

[70] Edgecombe GD, Giribet G (2003) Relationships of Henicopidae (Chilopoda: Lithobiomorpha): New molecular data, classification and biogeography*. African Invertebr 44: 13–38.

[71] Giribet G, Carranza S, Riutort M, Baguñà J, Ribera C (1999) Internal phylogeny of the Chilopoda (Myriapoda, Arthropoda) using complete 18S rDNA and partial 28S rDNA sequences. Philos Trans R Soc Lond B Biol Sci 354: 215–222 10.1098/rstb.1999.0373 10087567 PMC1692478

[72] Giribet G, Edgecombe GD (2006) Conflict between datasets and phylogeny of centipedes: an analysis based on seven genes and morphology. Proc Biol Sci 273: 531–538 10.1098/rspb.2005.3365 16537123 PMC1560052

[73] Giribet G, Edgecombe G (2006) The importance of looking at small-scale patterns when inferring Gondwanan biogeography: a case study of the centipede Paralamyctes (Chilopoda, Lithobiomorpha, Henicopidae). Biol J Linn Soc 89: 65–78.

[74] Vignal A, Milan D, SanCristobal M, Eggen A (2002) A review on SNP and other types of molecular markers and their use in animal genetics. Genet Sel Evol 34: 275–305 10.1051/gse 12081799 PMC2705447

[75] Palo J, Varvio SL, Hanski I, Väinölä R (1995) Developing microsatellite markers for insect population structure: complex variation in a checkerspot butterfly. Hereditas 123: 295–300. 8675443 10.1111/j.1601-5223.1995.00295.x

[76] Neff BD, Gross MR (2001) Microsatellite evolution in vertebrates: inference from AC dinucleotide repeats. Evolution 55: 1717–1733. 11681728 10.1111/j.0014-3820.2001.tb00822.x

[77] Aline F, Klank C (2010) Molecular Methods: Blessing or Curse. In: Habel JC, Assmann T, editors. Relict Species - Phylogeography and Conservation Biology. Berlin Heidelberg: Springer. pp. 309–320.

[78] Dányi L (2006) Faunistical research on the chilopods of Hungarian Lower Mountains. Nor J Entomol 53: 271–279.

[79] Varga Z (2003) Post-glacial dispersal strategies of Orthoptera and Lepidoptera in Europe and in the Carpathian basin. Proc 13th Int Coll EIS 105: 93–105.

